# A Novel Chondroitin AC Lyase With Broad Substrate Specificity From *Pedobacter rhizosphaerae*: Cloning, Expression, and Characterization

**DOI:** 10.3389/fbioe.2021.808872

**Published:** 2021-12-23

**Authors:** Li-Jian Zhou, Li-Bin Guo, Wei Wei, Zhi-Xiang Lv, Ye-Wang Zhang

**Affiliations:** ^1^ The People’s Hospital of Danyang, Affiliated Danyang Hospital of Nantong University, Danyang, China; ^2^ School of Pharmacy, Jiangsu University, Zhenjiang, China; ^3^ Zhongshiduqing Biotechnology Co. Ltd., Heze, China

**Keywords:** chondroitin AC lyase, cloning, characterization, expression, molecular docking

## Abstract

Chondroitin AC lyase (ChSaseAC) is one of the essential polysaccharides lyases in low molecular chondroitin sulfate production. In this work, a novel PrChSaseAC from *Pedobacter rhizosphaerae* was successfully cloned, expressed in *Escherichia coli*. After optimizing the induction, the recombinant PrChSaseAC could be expressed efficiently at 0.1 mM IPTG, 25°C, and 12 h induction. Then, it was purified with Ni-NTA affinity chromatography. The characterization of the purified PrChSaseAC showed that it had high specific activity and good storage stability, which would favor the production of low molecular weight chondroitin sulfate. It also displayed activity toward chondroitin sulfate C and hyaluronic acid. PrChSaseAC had the highest activity at pH 7.5, 37°C, 10 mM Ca^2+^, and 5 mg/ml of chondroitin sulfate A. Molecular docking of substrate and enzyme showed the interactions between the enzyme and substrate; it revealed that the enzyme showed high activity to CS-A and hyaluronic acid, but lower activity to CS-C attributed to the structure of the binding pocket. The high stability and specific activity of the enzyme will benefit the industrial production or clinical treatment.

## 1 Introduction

Chondroitin sulfate (CS) belongs to the glycosaminoglycan family formed with linear polysaccharides with repeated disaccharide units consisting of hexuronic acid and hexosamine (Mizumoto et al., 2013; Bhotmange and Singhal, 2015). It has been reported to have many biological and physiological functions. For example, it can post-transcriptionally modify CS proteoglycans acting as the critical modulators of neuronal plasticity, long-term memory, and neurodegenerative and psychiatric disorders (Yang, 2020). It was also found to be involved in essential life processes such as neurite outgrowth, virus infection, and metastasis (Lopez-Alvarez et al., 2020). CS’s primary usage in the clinic is in combination with glucosamine to treat rheumatism and osteoarthritis as anti-inflammatory medicine (Restaino et al., 2019). Thus, the demand for CS worldwide keeps increasing dramatically recently due to the increasing aging population.

However, CS’s drawbacks are its complicated structure of different positions and the number of sulfate groups due to the sources. According to the previous report, there are more than 25 CSs from various sources, and the molecular weight will directly influence the bioavailability (de Souza et al., 2018). Compared with CS, low molecular weight CS (LMWCS) has significant advantages, including higher bioavailability, better neuroprotective properties, and broader treatment in the clinic. Thus, the preparation of LMWCS received much attention in industrial production. Generally, LMWCS could be prepared by chemical degradation using HCl, alkaline or oxidative method, physical cleavage like microwave-assistant treatment, and enzymatic hydrolysis using chondroitin lyases. In these methods, enzymatic preparation is the most potential for environmentally friendless, highly specific, and mild reaction conditions (Li et al., 2016; Lan et al., 2020). The biocatalysts in the process, such as chondroitin lyase AC, ABC, and B, play essential roles in the preparation. These enzymes are classified on the basis of different substrate preferences. As a broad-spectrum enzyme, chondroitin lyase ABC (ChSase ABC) can catalyze the cracking of chondroitin sulfate A (CS-A), chondroitin sulfate C (CS-C), and dermatan sulfate (DS) through the β-elimination reaction model (Song et al., 2021). Chondroitin lyase AC (ChSaseAC) can degrade CS-A and CS-C, while chondroitin lyase B (ChSase B) can only degrade DS (Denholm et al., 2001). They are used individually or in combination at specific conditions for different requirements. To obtain highly stable LMWCS, the enzyme with high activity, specific activity, and stability is the high priority in the process.

ChSaseAC is one of the crucial enzymes in the production of LMWCS. It was first isolated from *Flavobacterium heparinum* by Payza and Korn (Payza and Korn, 1956). Later, it was also found and identified in other bacteria, including *Bacteroides thetaiotaomicron* (Linn et al., 1983), *Arthrobacter aurescens* (Hiyama and Okada, 1975, Hiyama and Okada, 1977), *Streptococcus intermedius* (Shain et al., 1996), and *Porphyromonas gingivalis* W50 (Smith et al., 1997). However, the expression and purification of the enzyme in the original organisms are quite tricky, which leads to low production. To improve the expression and purification, several ChSases AC from *F. heparinum* (Tkalec et al., 2000) and *Arthrobacter* sp. SD-04 (Chen et al., 2019) have been reported to have a heterologous expression in *Escherichia coli*, but the target protein was expressed mostly in insoluble form. Moreover, their highest specific activities were 46.9 and 0.9 U/mg, respectively, when the substrate was CS-A. Therefore, these ChSases AC still suffer low activity and stability.

In this work, a novel ChSaseAC from *Pedobacter rhizosphaerae* was cloned, expressed, and characterized. The expression was optimized to get high enzyme production. The characterization of the ChSaseAC showed that it has good storage stability and broad substrate spectra. The structure of the enzyme was investigated with molecular docking to explore the interaction with the substrates.

## 2 Results

### 2.1 Sequence Alignment and Identification of PrChSaseAC

**FIGURE 1:**
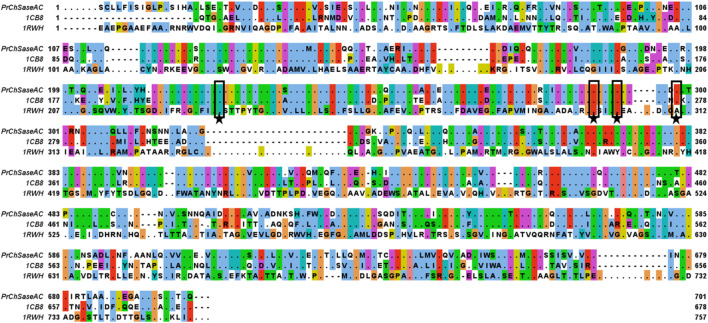
Multiple-sequence alignment of the ChSaseAC from *F. heparinum* (1cb8), *P. rhizosphaerae* (PrChaseAC), and *A. aurescens* (1rwh). The residues of PrChSaseAC and the non-conserved residues of other ChSaseAC were shown, and it was generated with Jalview. The residues of active sites were marked with stars.

The full length of the encoding ChSaseAC gene from *P. rhizosphaerae* was 2,025 bp and encoded 675 amino acids. The sequences of ChSaseACs from different organisms were aligned and are presented in Figure 1. PrChSaseAC shows more than 60% identity to FhChSase, which means that they have high homology. However, there is only 23% identity to AaChSase, indicating that they are derived from different ancestors. According to the previous report (Féthière et al., 1999), the residues His225, Arg288, Arg292, and Lys298 might be involved in the binding to the substrate and contribute to the catalysis residues highly conserved compared to the template 1cb8. Although the similarity of PrChSaseAC to AaChSaseAC is only 22%, the steric structure is highly similar, especially the secondary elements like *α*-helix, *β*-sheet, and *β*-turn. The sequencing results of the recombinant PrChSaseAC proved that the enzyme was cloned successfully.

### 2.2 Expression and Purification of PrChSaseAC

**FIGURE 2:**
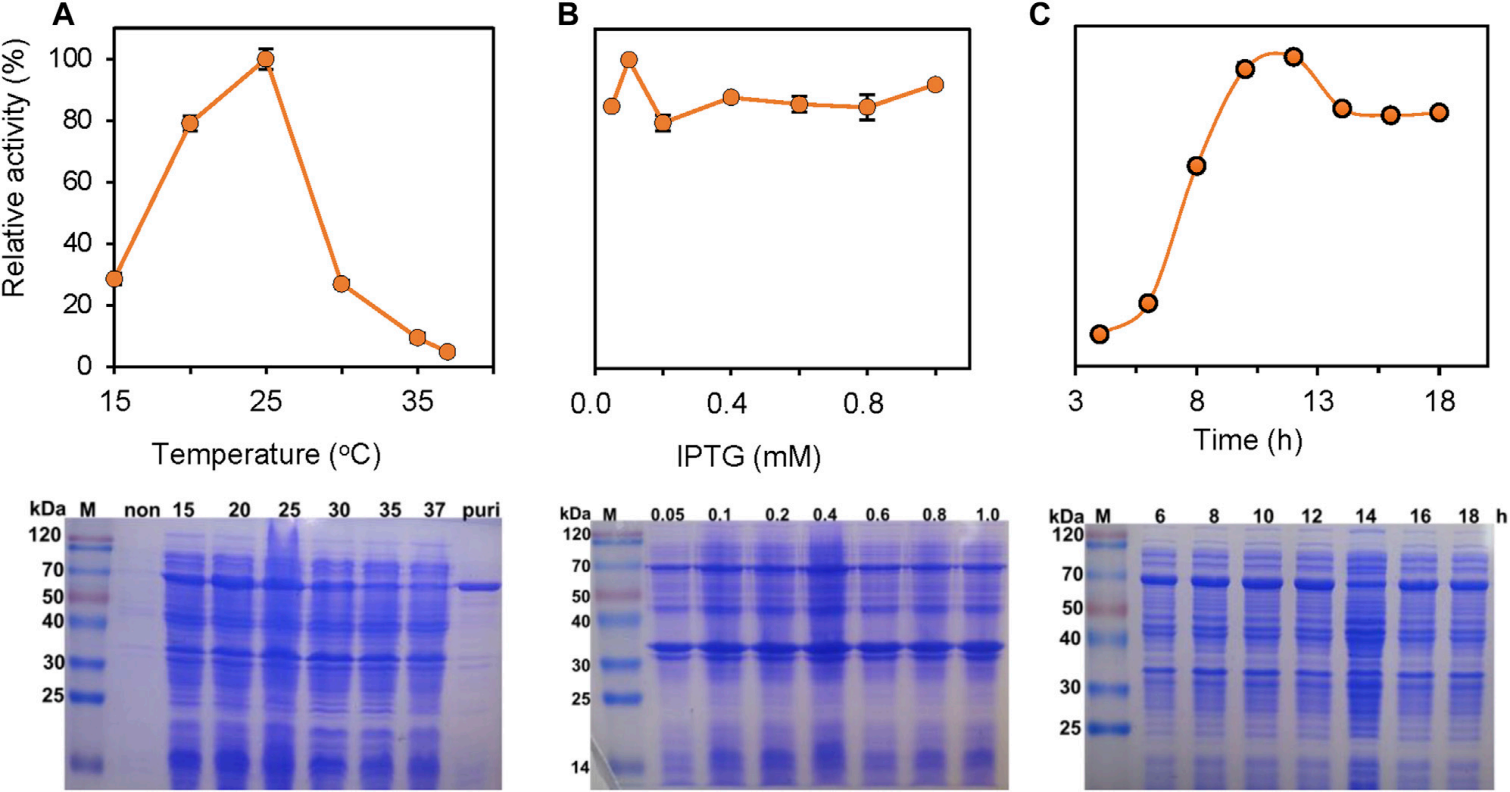
Effects of the induction temperature **(A)**, concentration of IPTG **(B)**, and induction time **(C)** on the enzyme activity. Insets are the SDS-PAGE images of the supernatant in the production of ChSaseAC: M: protein molecular weight marker; Puri: the purified enzyme.

The expression of PrChSaseAC in *E. coli* BL21 (DE3) was investigated in different induction conditions, shown in Figure 2. The enzyme activities were determined, and the expression was observed with SDS-PAGE. The induction temperatures were varied from 15°C to 37°C under the conditions of 0.2 mM isopropyl-*β*-D-thiogalactopyranoside (IPTG) and 7 h induction. As shown in Figure 2A, the highest activity was obtained at 25°C. Moreover, enzyme activity could not be detected when the induction temperature was increased to 37°C. Figure 2B shows the effect of the IPTG concentration on soluble expression. Although there was not much change of the enzyme activity when the concentration of IPTG was varied from 0.05 to 1.0 mM, the highest enzyme activity could be achieved at 0.1 mM IPTG. Figure 2C shows the effect of induction time on the expression of PrChSaseAC. When increasing the induction time from 4 to 10 h, the enzyme activity was improved from 10% to 96% and reached the highest activity at 12 h.

PrChSaseAC was heterologously expressed in *E. coli* BL21 (DE3) as an N terminal 6×His-tagged protein. The recombinant enzymes were further purified by affinity chromatography with the Ni-NTA column. SDS-PAGE analysis showed that PrChSaseAC was successfully purified with the molecular weight of about 77 kDa (Figure 2A). Furthermore, the highest activity of 361.4 U/mg was obtained under the induction conditions of 0.1 mM IPTG, 20°C, and 12 h.

### 2.3 Biochemical Characterization of PrChSaseAC

**FIGURE 3:**
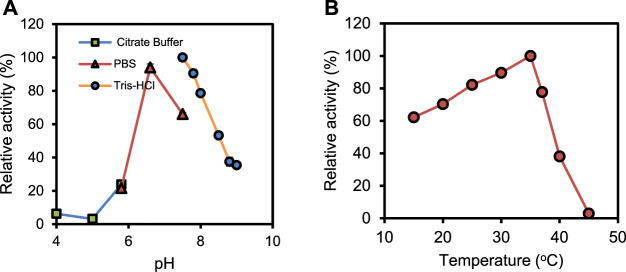
Effects of pH **(A)** and temperature **(B)** on the activity of PrChSaseAC. The enzyme activity was measured according to the enzyme assay, and every experiment was repeated three times.

The biochemical characterization of the recombinant PrChSaseAC was performed, and the data are shown in Figure 3. Figure 3A shows the effect of pH on the activity of PrChSaseAC. The enzyme displayed high activity (>80%) at the pH range from 6.5 to 8.0. Moreover, the enzyme activity was significantly affected by different buffers. Tris–HCl buffer could be the most suitable buffer for the hydrolysis of CS-A. In Figure 3B, the effect of temperature on the enzyme activity was investigated ranging from 15°C to 45°C. The enzyme activity was increased from 62% to 100% when the temperature was increased from 15°C to 37°C. However, it decreased rapidly when the temperature was continued to increase, and no activity was detected at 45°C.

**FIGURE 4:**
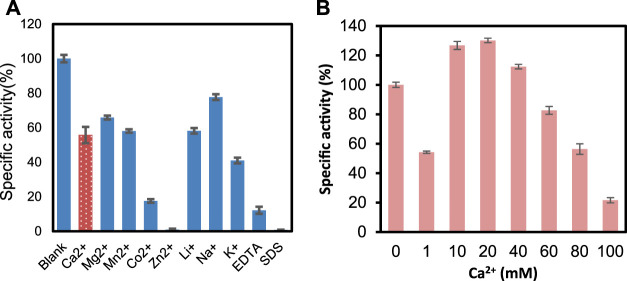
**(A)** Metal ions EDTA and SDS on the activity of PrChSaseAC. **(B)** Concentration of Ca^2+^ on the activity of PrChSaseAC.

Figure 4A presents the effects of metal ions, EDTA, and SDS on the enzyme activity. At 1 mM concentration, there is not much change in the activity when adding Na^+^, K^+^, and Ca^2+^. However, the enzyme was inhibited by Zn^2+^, SDS, and EDTA significantly. It was reported that Ca^2+^ plays an essential role in ChSase-catalyzed reactions (Guo et al., 2021). PrChSaseAC showed Ca^2+^-dependent catalytic properties, but high Ca^2+^ concentrations inhibited the enzyme activity (Figure 4B). This may be that the excess Ca^2+^ in the reaction mixture plays the role of a competitive inhibitor which leads to unfavorable conformation and prevents the substrate binding.

**FIGURE 5:**
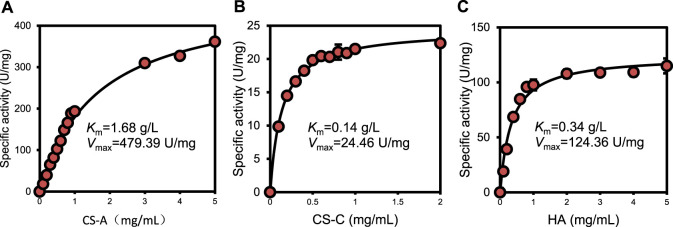
Effect of substrate concentration on the activity of ChSaseAC **(A)** CS-A, **(B)** CS-C, and **(C)** HA.

Kinetic parameters were determined for recombinant ChSaseAC against CS-A, CS-C, and HA substrates. Figure 5 shows the effects of the concentrations of three substrates (CS-A, CS-C, and HA) on the enzyme activity. The highest specific activity of 361.4 U/mg was obtained when the CS-A concentration was 5 mg/ml. The *K*_*m*_ and *V*_max_ against CS-A were calculated as 1.68 g/l and 479.39 U/mg with nonlinear regression, respectively. PrChSaseAC has a *K*_*m*_ of 0.14 g/l and a *V*_*max*_ of 24.46 U/mg using the CS-C as the substrate. The *K*_*m*_ and *V*_*max*_ toward HA were 0.34 g/l and 124.36 U/mg, respectively. Besides, PrChSaseAC also showed specific activities of 21.5 and 97.6 U/mg to CS-C and HA, respectively. The comparison of ChSaseACs from different bacteria is presented in table 1. It can be seen that PrChSaseAC has broad substrate specificity and can catalyze CS-A, CS-C, and HA. PrChSase has the highest V_max_ (479.39 U/mg), which is 6.2, 10, and 529 folds those of chondroitin lyases AC from *B. stercoris*, *F. heparinum*, and *Arthrobacter sp.*, respectively.

**TABLE 1:** Comparison of ChSaseACs’ kinetic parameters from different bacteria.

	Substrates						References
	CS-A		CS-C		HA		
Organisms	*K*_*m*_ (mg/mL)	*V*_*max*_ (U/mg)	*K*_*m*_ (mg/mL)	*V*_*max*_ (U/mg)	*K*_*m*_ (mg/mL)	*V*_*max*_ (U/mg)	
*Arthrobacter sp*	0.971	0.905	0.334	0.442	NA	NA	Chen et al. (2019)
*F. heparinum*	NA	46.9	NA	31.3	NA	NA	Tkalec et al. (2000)
*B. stercoris*	0.39	76.7	0.16	29.2	NA	NA	Shim and Kim (2008)
*P. rhizosphaerae*	1.68	479.39	0.14	24.46	0.34	124.36	This work

NA, not available.

### 2.4 The Storage Stability of PrChSaseAC

**FIGURE 6:**
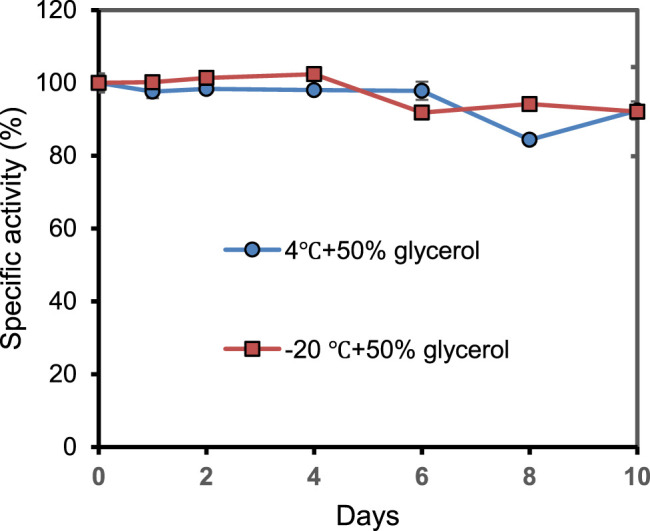
The storage stability of PrChSaseAC at 4°C and −20°C. The enzyme was stored in pH 7.5 Tris–HCl buffer containing 50% glycerol.

As shown in Figure 6, the storage stability of PrChSaseAC was investigated at 4°C and −20°C. The enzyme displayed high stability at the storage conditions and kept more than 92% residual activity after 10 days.

### 2.5 Homology Modeling and Substrate Docking

**FIGURE 7:**
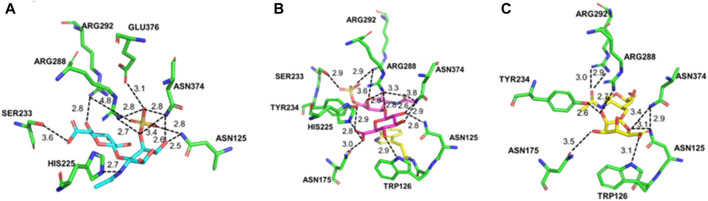
Molecular docking of PrChSaseAC and CS-A **(A)**, CS-C **(B)**, and HA **(C)**.

The interactions between the enzyme and substrates, including CS-A, CS-C, and HA, are shown with PyMOL in Figure 7. As shown in Figure 7A, Asn125, His225, Ser233, Arg288, Arg292, Asn374, and Glu376 were found to bind with CS-A to form hydrogen bonds. There are 11 hydrogen bonds formed in total between the catalytic residues with the disaccharide. A hydrogen bond of 2.7 Å was formed between the imidazole of His225 and the N atom of the disaccharide’s acetyl group. Asn374, Arg288, and Arg292 formed 7 hydrogen bonds with the substrate. Compared with the CS-A-enzyme complex, there were 14 hydrogen bonds formed between CS-C and the enzyme. An exception of the residues mentioned above involving the binding of CS-C, Trp126, Asn175, and Tyr234 also involved the substrate binding (Figure 7B). In the complex of the HA-enzyme, there were only 7 hydrogen bonds formed between the substrate and the enzyme (Figure 7C). Ser233 and His225 do not involve HA’s binding compared to the binding with CS-A and CS-C.

## 3 Discussion

Chondroitin AC lyase, also named chondroitinase AC, is the secondary family of polysaccharide lyases. It can depolymerize glycosaminoglycans by eliminating a 4-linked hexosamine, resulting in an unsaturated C4–C5 bond on the hexuronic acid moiety. Although several ChSasesAC have been identified or heterologously expressed (Chen et al., 2019), they cannot meet industrial production requirements. Thus, searching for novel enzymes with high activity and stability is still practicing the increasing market of LMWCS.

In the present work, a novel PrChSaseAC was successfully cloned, expressed in *E. coli*. For heterologous expression, the IPTG concentration, induction temperature, and time were optimized to obtain high production of the enzyme. The expressed enzyme with a 6×His tag could be readily purified by affinity chromatography. After purification, the enzyme’s characterization was investigated, including the effects of pH, temperature, metal ions, and substrate concentration on the activity. The enzyme displayed the highest activity of 361.4 U/mg in 20 mM of pH 7.5 Tris–HCl buffer at 37°C, which was 7.7-fold and 401.6-fold higher than that from *F. heparinum* and *Arthrobacter* sp. SD-04, respectively. In the characterization of metal ions, it is worth noting that low-concentration Ca^2+^ inhibited a specific activity, while high concentration enhanced the specific activity. This might be that the enzyme has two or more Ca^2+^-binding sites; some conformational changes after the binding might inhibit the activity, while more favorable conformational changes were dominated after the binding. These results indicated that the enzyme could work efficiently in phycological conditions, which will benefit the production of LMWCS or the clinic’s applications. Compared with the previous work on the ChSaseAC, this ChSase could catalyze the biotransformation of CS-A, CS-C, and HA. It could be stored at 4°C or −20°C, and there was only 8% specific activity lost after 10 days. It indicated that PrChSaseAC showed apparent advantages in the broad substrate specificity and storage stability, which will be conducive to the industrial production. Also, the immobilization in the future could improve the stability of the enzyme (Li et al., 2015; Xu et al., 2018; Zhuang et al., 2018; Xu et al., 2020; Fan et al., 2021).

Molecular docking was performed to validate the substrate specificity and the specific activity to explore the enzyme and substrate interaction. PrChSaseAC showed high activity to CS-A and HA, respectively. Molecular docking showed that more hydrogens were formed in the active pocket among ChSaseAC-CS-C, which limits the extension of the substrate in the active center, resulting in lower enzyme activity (Li et al., 2019, Li et al., 2020; Su et al., 2022), while HA lacks the sulfate moieties of galactosamine, resulting in the decreased ability on the recognition of substrate for enzyme, which makes the enzyme activity slightly lower than using CS-A as the substrate. Asn125, Arg288, Arg292, and Asn374 are involved in the binding of the substrate. His255 was reported to be the catalytic residue for the cleavage of the glycoside bond of CS. According to the previous work, Ser233 was one of the O-glycosylation sites (Féthière et al., 1999), and it could interact with the carboxyl group or sulfate of the disaccharide. Asn125, Arg288, Arg292, and Asn374 would help stabilize the complex’s conformation, lowering the reaction’s free energy. Trp126 could form a hydrogen bond with CS-C or HA, which could also stabilize the substrate–enzyme complex conformation.

## 4 Materials and Methods

### 4.1 Materials

DNA extraction kits, DNA polymerase for PCR, and T4 DNA ligase were purchased from Beyotime Biotech Co. Ltd. (Haimen, China). The PET-28(a) expression plasmid, restriction endonucleases, oligonucleotide primers, and competent cells, including *E. coli* DH5α and BL21 (DE3), were provided from Sangon Bioengineering Co. Ltd. (Shanghai, China). A nickel-nitrilotriacetic acid (Ni-NTA) superflow column was supplied by TransGen Biotech Co. Ltd. (Beijing, China). The DNA ladder and protein molecular weight marker were obtained from Detai Biologics Co. Ltd. (Nanjing, China). CS-A from the bovine trachea, CS-C from shark cartilage, and hyaluronic acid (HA) sodium salt from *Streptococcus equi* were purchased from Sigma Co. Ltd. (St. Louis, MO, USA). Kanamycin sulfate, IPTG, and all other reagents used in the experiments were of analytical grade and ordered from Aladdin Co. Ltd. (Shanghai, China).

### 4.2 Strains and the Culture Conditions

The original strain *P. rhizosphaerae* was supplied by the Guangdong Microbiology Culture Center (Guangzhou, China) and cultured at 37°C in brain heart infusion (BHI) medium. The competent cells *E coli* strains DH5α and BL21 (DE3) were cultivated in Luria–Bertani (LB) broth containing 50 μg/ml kanamycin sulfate at 37°C.

### 4.3 Enzyme Assay

The enzyme assay for ChSaseAC was performed with an ultraviolet spectrophotometer at 232 nm, according to our previous report (Liu et al., 2020; Guo et al., 2021). The enzymatic reaction mixture consisted of 890 μl of 20 mM, pH 7.5 Tris–HCl buffer containing 10 mM CaCl_2_, 100 μl of 5 mg/ml CS-A, and 10 μl of enzyme solution (∼10 μg). Moreover, the reaction was performed at 30°C. To check the enzyme activity for CS-C or HA, the enzymatic reaction was carried out under the same conditions except that CS-A was replaced by CS-C or HA. The activity of the enzyme was calculated by monitoring the UV absorbance at 232 nm. Moreover, the molar extinction coefficient of the unsaturated uronic acid is 5,500 l/(mol cm). One international unit was defined as the amount of protein that can produce 1 μmol unsaturated uronic acid per minute at 30°C.

### 4.4 Cloning, Expression, and Purification of the Chondroitin AC Lyase

*P. rhizosphaerae* genome DNA was extracted using the DNA extraction kits, and it was used as the template for amplification of the ChSaseAC gene by polymerization chain reaction (PCR). The primer oligonucleotides were listed as follows: 5′-CGG​GAT​CCG​AAA​CCA​CAA​AAG​TGA​TCA​TGG-3′ and 5′-AAC​CGT​CAC​GCT​TCA​ATA​ACT​CGA​G-3′ (*BamH* I and *Xho* I restriction sites are underlined, respectively). After amplification, the amplified product was digested with the *BamH* I and *Xho* I endonucleases. Then, it was ligated with pET-28a, which was digested with the same restriction enzymes and recovered from the agarose gel. The recombinant plasmid of pET-28a-ChSaseAC was then transformed into *E. coli* DH5α and then grown in LB plates containing kanamycin sulfate (50 μg/ml) and cultured at 37°C. DNA sequencing was used to confirm positive transformants.

The recombinant plasmids were transformed into *E. coli* BL21 (DE3) cells. The recombinant strains were cultured in LB with kanamycin (50 μg/ml) at 37°C until the OD_600_ value reached 0.8. Then, the recombinant strains were incubated with 0.1 mM IPTG at 25°C for 12 h. The cells were disrupted by a high-pressure cracker in an ice-cold environment. The supernatant was obtained by centrifugation. A Ni-NTA column was used to purify the protein. The purity of the enzyme was analyzed using 12% SDS-PAGE. Coomassie Brilliant Blue R-250 was used to stain the proteins in the gels. Protein concentrations were determined by the Bradford method.

### 4.5 Biochemical Characterization of the Chondroitin AC Lyase

For the characterization of the recombinant ChSaseAC, several factors influencing enzyme activity were investigated. The effect of pH on the activity of the recombinant ChSaseAC was examined in 20 mM citrate buffer (pH 4.0–6.0), phosphate buffer (pH 6.0–7.5), and Tris–HCl buffer (pH 7.5–9.0) (Guo et al., 2021). For the effect of temperature, the activities of ChSaseAC at a temperature range of 15°C–45°C were measured. The effects of metal ions SDS and EDTA on the activity of ChSaseAC were checked in Tris–HCl buffers containing 1 mM MgCl_2_, MnCl_2_, ZnCl_2_, CoCl_2_, CaCl_2_, LiCl, NaCl, KCl, SDS, and EDTA, respectively. Besides, the influence of the concentrations of Ca^2+^ on enzyme activity of ChSaseAC was studied. The reaction buffer without metal ions was set as the control.

The kinetic parameters (*K*_*m*_ and *V*_*max*_) of the recombinant ChSaseAC for different substrates (CS-A, CS-C, and HA) were determined using a series of substrate solutions (0.01–5 mg/ml) under the conditions of 35°C and pH 7.5. They were calculated with nonlinear regression of the Michaelis–Menten equation. All the experiments were repeated 3 times.

### 4.6 The Storage Stability of the Chondroitin AC Lyase

The chondroitin AC lyase’s storage stability was investigated in 20 mM of pH 7.5 Tris–HCl buffer with 50% glycerol at 4°C and −20°C. After certain intervals, the stored enzyme was withdrawn and subjected to enzyme assay to evaluate the specific and relative activity. Then, t_1/2_ was calculated with linear regression.

### 4.7 Homology Modeling and Substrate Docking

Protein sequence analysis showed that PrChSaseAC from *P. rhizosphaerae* shared 64.3% identity with ChSaseAC from *F. heparinum*, and multiple-sequence alignment of three ChSaseACs was performed with ClustalX (Chenna et al., 2003) and shown with Jalview (2.11.1.2) (Waterhouse et al., 2009). A three-dimensional model structure of PrChSaseAC was built using the automated protein structure homology modeling server SWISS-MODEL (Bordoli and Schwede, 2012) according to the known crystal structure of ChSaseAC from *F. heparinum* (PDB: 1cb8). The reliability of the model was verified by PROCHECK (Laskowski et al., 1993). Adding of polar hydrogens, assigning of Gasteiger charges to all atoms of the disaccharide, and docking of the substrate into the active site of ChSase were all performed with AutoDock4 (Morris et al., 2009). All the structural representations were generated using PyMOL (2.5.0) (Seeliger and de Groot, 2010).

## 5 Conclusion

In summary, a stable novel ChSase-AC was cloned, expressed, and characterized. The enzyme properties showed that it could be applied in the production of LMWCS or the clinic for its stability and broad substrate specificity. The molecular docking of the substrates, including CS-A, CS-C, and HA, revealed that the enzyme could catalyze CS-A and CS-C more efficiently than HA because of the binding pocket structure. To further improve the catalytic efficiency, protein engineering of the enzyme could be performed in the future.

## Data Availability

The original contributions presented in the study are included in the article/supplementary material; further inquiries can be directed to the corresponding author.
